# Career Satisfaction and Perceived Salary Competitiveness among Individuals Who Completed Postdoctoral Research Training in Cancer Prevention

**DOI:** 10.1371/journal.pone.0169859

**Published:** 2017-01-25

**Authors:** Jessica M. Faupel-Badger, David E. Nelson, Grant Izmirlian

**Affiliations:** 1 National Institute of General Medical Sciences, Bethesda, Maryland, United States of America; 2 Division of Cancer Prevention, National Cancer Institute, Bethesda, Maryland, United States of America; University of California Irvine, UNITED STATES

## Abstract

Studies examining career satisfaction of biomedical scientists are limited, especially in the context of prior postdoctoral training. Here we focused on career satisfaction defined as satisfaction with one’s career trajectory and perceived salary competitiveness among a predominantly Ph.D.-trained population of scientists who completed cancer prevention-related postdoctoral training between 1987–2011. National Cancer Institute (NCI) Cancer Prevention Fellowship Program (CPFP) alumni (n = 114), and previous recipients of NCI-sponsored Ruth L. Kirschstein National Research Service Award (NRSA/F32) postdoctoral fellowships (n = 140) completed online surveys. Associations of career satisfaction and perception of salary competitiveness with demographic, training, and employment-related factors were examined using logistic regression. Overall, 61% reported high levels of satisfaction with their career trajectory to-date. Higher salary (odds ratio [OR] = 2.86, 95% confidence interval [95% CI]: 1.07–7.69) and having more leadership roles (OR = 2.26, 95% CI:1.04–4.90) were independently associated with higher career satisfaction. Persons with race/ethnicity other than white (OR = 0.40, 95% CI: 0.20–0.82) or age ≥ 50 (OR = 0.40, 95%CI: 0.17–0.94) had lower career satisfaction levels. There were no statistically significant differences in career satisfaction levels by gender, scientific discipline, or employment sector. 74% perceived their current salary as competitive, but persons with 5–9, or ≥10 years in their current position reported lower levels (OR = 0.31, 95% CI: 0.15–0.65; and OR = 0.37, 95% CI: 0.16–0.87, respectively), as did individuals in government positions (OR = 0.33, 95% CI: 0.11–0.98). These data add to the understanding of career satisfaction of those with advanced training in biomedical research by examining these measures in relation to prior postdoctoral research training and across multiple career sectors.

## Introduction

Numerous reports have highlighted the dearth of information about the careers of former postdoctoral research fellows in the biomedical sciences [1–5]. Research on this population has emphasized scientific productivity (e.g., grants or publications), often examining advancement of scientists employed in academic or other research institutions to tenured positions [6–14]. Such data are important, but focus on only a narrow aspect of possible career outcomes and may not be applicable to those employed outside of academic positions.

Although not restricted to former postdoctoral fellows, current job satisfaction and current salary, especially gender differences, are other outcomes that have been studied. Overall, they indicate that biomedical scientists are very satisfied with their current jobs [11, 15]. But studies of physicians [16–18] or predominantly PhD-educated academic science faculty members [6, 13, 19, 20, 21] generally find that women have lower current job satisfaction and salary levels than their male counterparts, although some gender differences are reduced or eliminated when analyses control for scientific discipline [13].

A broader issue that has rarely been researched is the career satisfaction of biomedical scientists when asked in the context of satisfaction with their career trajectory to-date [22, 23] rather than satisfaction with their current job. Career satisfaction is a retrospective assessment of how pleased individuals are with their choice of, and experiences within, the careers of their choosing across the individual jobs they have held [22, 23]. To our knowledge, there have been no studies of former postdoctoral fellows and their level of career trajectory satisfaction, or of whether they perceive themselves to be fairly compensated for their work. This seems surprising because individuals who complete doctoral education, and subsequent postdoctoral training, in the biomedical sciences devote many years, and often significant personal financial resources, to prepare for employment in their field [24–26].

We recently conducted a survey of scientists who completed postdoctoral training between 1987–2011 in the field of cancer prevention, either at the National Cancer Institute (NCI) [27, 28], or at an array of research institutions by virtue of receiving individual NCI postdoctoral fellowship awards [29]. As part of this survey, data were collected on career satisfaction and perceived salary competitiveness from biomedical scientists across a range of scientific disciplines, and who were currently employed in different career sectors. These data from former postdoctoral fellows provided us a unique opportunity to comprehensively examine the (1) overall levels of career satisfaction and perceived salary competitiveness; and the (2) potential independent association of other factors with these measures, including scientific discipline, demographics, time since completing postdoctoral training, and employment or job activity factors.

## Methods

Data for this study came from an on-line survey of individuals who completed postdoctoral traning between 1987–2011 either in the NCI Cancer Prevention Fellowship Program (CPFP) [27, 28] or were recipients of a Ruth L. Kirschstein National Research Service Award (NRSA/F32) [29] individual-level postdoctoral training grant and completed research fellowships in research institutions throughout the United States related to cancer prevention (http://www.cancer.gov/grants-training/training/funding/f32). Extensive details about the survey and sample methodology are provided elsewhere [27, 30].

Briefly, the CPFP has trained early career scientists from multiple disciplines at NCI facilities for nearly 30 years. The program admits 10–15 individuals annually through a competitive process, and funding support is available for up to four years. This is a structured, postdoctoral training program that utilizes a cohort model and has defined curriculum components [28]. Eligible NRSA/F32 participants were individuals who conducted cancer prevention-related postdoctoral research at different institutions around the United States. To identify individuals who conducted such research, we used the following search terms: cancer prevention; vaccine; cancer risk; chemoprevention; antitumor; nutrition; tumor suppressor; carcinogenesis; lifestyle factors; environmental factors; interventions; risk factors; personal behaviors; cancer screening; infections; tobacco; cancer control; occupational factor; early detection; or physical activity. In contrast to the CPFP alumni, NRSA/F32 awardees are individually funded and will be participating in professional development opportunities as part of individualized, tailored training plans rather than as a cohort model. Although these two populations had different training experiences, both populations were competitively selected for their postdoctoral funding and elected to focus on cancer prevention-related topics in their postdoctoral training.

All participant survey activities were conducted by Westat in 2014. A total of 205 CPFP alumni and 362 NRSA/F32 recipients met eligibility criteria. Potential participants were initially notified by email about the survey, followed two weeks later with a mailed letter. Subsequent reminder emails or phone calls or were done as necessary with non-responders. Surveys were completed by 123 CPFP alumni (59% response rate) and 146 NRSA/F32 recipients (39% response rate). No individual identifier information was included in the final dataset, and specific demographic categories were irreversibly combined (e.g., race other than white) to avoid cell sizes possibly leading to individual respondent identification. NIH and Westat Institutional Review Boards independently reviewed the study design and materials, and deemed it exempt under rule 45 CFR 46.101(b) (2).

The survey instrument was developed based on prior scientific literature and previous studies conducted by NCI and Westat [8, 30]. The level of career satisfaction was assessed using the question: “How satisfied are you with the progression of your career to this point?” Potential response categories were extremely satisfied, very satisfied, somewhat satisfied, a little satisfied, and not at all satisfied. Perceived salary competiveness was based on a yes or no response to the question: “Do you feel that your salary is generally competitive with others in similar positions?” We restricted analyses to respondents who had completed postdoctoral training and were currently employed (total n = 254).

### Statistical Analyses

All analyses were conducted using R, version 3.1.2 [31]. For logistic regression analyses, career satisfaction was dichotomized as “extremely satisfied or very satisfied” vs. “somewhat satisfied, a little satisfied, or not at all satisfied,” and perceived salary competitiveness as “yes” vs. “no.” Preliminary findings indicated a strong correlation between career satisfaction and perceived salary competitiveness, so analyses were conducted separately for these as dependent variables.

We analyzed career satisfaction and perceived salary competitiveness by the following variables: age group (<40, 40–49, ≥50 years), sex, fellowship cohort based on quartiles (1987–1996, 1997–2001, 2002–2006, 2007–2011), race/ethnicity (White non-Hispanic, other), type of doctoral degree (Ph.D., other doctoral degree), cancer prevention postdoctoral training (CPFP, F32/NRSA), total number of postdoctoral training positions (1, >1), current salary (<$75,000; $75,000-$99,999; $100,000-$124,999; $125,000-$149,999; $150,000-$174,999; ≥$150,000), current employer (university or other research institution, government agency, other employer), length of time in current position (<5, 5–9, ≥10 years), and percent of time conducting research-related activities in current position (none, 1%-25%, 26%-50%, 51%-75%, ≥76%).

The number of career leadership roles in professional activities since completing postdoctoral training was based on reporting being involved to a large extent, or a very large extent, in one of five cancer research activities: pursuing a new theoretical direction or addressing a previously unexplored topic; making a significant contribution to a scientific breakthrough; making a significant contribution to advance innovative ideas; addressing key knowledge gaps; or developing funding initiatives to address knowledge gaps. The scientific discipline categories for former fellows were developed based on principal components analyses because the survey allowed binary (yes/no) responses to eight separate discipline questions with overlap allowed. Loadings from the first principal component indicated two discipline indicator variables would suffice: social science/epidemiology vs. other, and biomedical research vs. other.

Separate bivariate associations were tabulated to assess the relationship between each independent variable with each dependent variable of interest using p-values obtained from chi-square or Fisher’s exact tests. Variables with ≥5 response categories were reduced to a smaller number of levels in these analyses by combining selected categories. Separate multiple logistic regression models were then created and analyzed for each dependent variable, with independent variables selected for inclusion based on p-values <0.25 from the separate bivariate associations. Odds ratios with 95% confidence intervals from multiple logistic regression models were used to assess the level of association of each independent variable with greater levels of career satisfaction, or a perception that salaries were competitive, after controlling for all other variables.

## Results

Basic descriptive characteristics of the population including CPFP alumni and NRSA/F32 award recipients are provided in Table 1. As noted in the methods, approximately 59% of CPFP alumni and 39% of F32 awardees who were sent the survey completed the instrument. There were limited data in which to compare non-responders to responders. For NRSA/F32 awardees only application year was available. For CPFP alumni, application year, age, gender and race were available.) Using Chi-square analysis, there were no statistically significant differences at p<0.05 between responders and non-responders (data not shown). The majority of respondents were women and white, non-Hispanics. Nearly three-quarters completed only one postdoctoral fellowship; more than 90% had a Ph.D. degree; and nearly half listed biomedical science as their scientific discipline. The majority were currently employed at a university or other research institute, with almost half in their current position for <5 years.

**Table 1:** Selected Characteristics of the Study Population.

Characteristic	n = 254 (%)
**Age (yrs)**	
**<40**	**34.2**
**40–49**	**44.7**
**≥50**	**21.1**
**Sex**	
**Male**	**36.4**
**Female**	**63.6**
**Race/Ethnicity**	
**White, non-Hispanic**	**78.8**
**Other**	**21.2**
**Salary ($)**	
**<75,000**	**17.1**
**75,000–99,999**	**22.2**
**100,000–124,999**	**27.0**
**125,000–149,999**	**13.1**
**150,000–174,999**	**9.9**
**≥175,000**	**10.7**
**Postdoctoral Population**	
**CPFP alumni**	**44.9**
**NRSA/F32 recipients**	**55.1**
**Postdoctoral Cohort**	
**1987–1996**	**15.4**
**1997–2001**	**28.3**
**2002–2006**	**29.9**
**2007–2011**	**26.4**
**Number of postdoctoral fellowships**	
**One**	**72.4**
**Two or more**	**27.6**
**Doctoral Degree**	
**PhD**	**91.6**
**Other**	**8.4**
**Scientific Discipline**	
**Biomedical science**	**46.9**
**Epidemiology**	**28.3**
**Behavioral/social science**	**15.0**
**Medicine**	**12.6**
**Nutrition science**	**7.1**
**Physical science**	**7.1**
**Mathematics**	**2.0**
**Other**	**12.2**
**Current Employer**	
**University/research institute**	**57.1**
**Government**	**27.2**
**Other**	**15.7**
**Years in Current Job**	
**<5**	**49.6**
**5–9**	**29.1**
**≥10**	**21.3**

*Total >100% because respondents could list more than one

Overall career satisfaction levels were high among this population, although there was a difference in the distribution of responses for CPFP alumni (greater satisfaction) compared with NRSA/F32 recipients (Table 2). Close to three-fourths in both groups perceived their current salary level as competitive.

**Table 2:** Career Satisfaction and Perceived Competitive Salary by Postdoctoral Cohort.

	CPFP Alumni n = 114 (%)	NRSA/F32 Recipients n = 140 (%)	p value
**Career Satisfaction Level**			
**Extremely satisfied**	**22.8**	**10.1**	
**Very satisfied**	**49.1**	**41.7**	
**Somewhat satisfied**	**24.6**	**34.5**	**0.002**^a^
**A little satisfied**	**3.5**	**10.8**	
**Not satisfied**	**0.0**	**2.9**	
**Perceived Competitive Salary**			
**Yes**	**71.9**	**76.3**	
**No**	**28.1**	**23.7**	**0.552**^b^

^a^ P value from Fisher’s exact test, significance at p<0.05;

^b^ P value from Χ^2^ test, significance at p<0.05.

The multiple logistic regression model for career satisfaction contained 10 variables (Table 3). Career satisfaction was lower among those aged ≥50 years and race/ethnicity other than white. In contrast, career satisfaction was higher among those with a salary ≥$150,000, or with 5 leadership roles. There was no association by fellowship cohort, sex, or current employer. In addition, there was no difference in employment sector by cohort as defined by entering the CPFP or receiving an NRSA/F32 award between 1987–1996, 1997–2001, 2002–2006, or 2007–2011 and this variable was not included in the multivariate model (data not shown).

**Table 3:** Relative Odds of Selected Factors for Career Satisfaction among Former Postdoctoral Fellows.

Factor	Odd Ratio and 95% CI^a^
Age	
<40 (referent)	1.00
40–49	1.08 (0.55–2.10)
≥50	0.40 (0.17–0.94)
Race/Ethnicity	
White, non-Hispanic	1.00
Other	0.40 (0.20–0.82)
Salary ($)	
<100,000 (referent)	1.00
100,000–149,999	1.84 (0.91–3.69)
≥150,000	2.86 (1.07–7.69)
Postdoctoral Population	
NRSA/F32 recipients (referent)	1.00
CPFP alumni	1.06 (0.46–2.45)
Doctoral Degree	
PhD (referent)	1.00
Other	1.82 (0.47–7.09)
Biomedical Science Discipline	
No (referent)	1.00
Yes	0.75 (0.37–1.52)
Social/Behavioral Science Discipline	
No (referent)	1.00
Yes	1.86 (0.84–4.13)
Time Spent in Research-Related Activities	
≥51% (referent)	1.00
26%-50%	1.34 (0.55–3.30)
≤25%	1.71 (0.66–4.44)
Current Employer	
Other (referent)	1.00
Government	1.12 (0.40–3.17)
University/research institute	0.78 (0.33–1.84)
No. of Leadership Roles	
0–2 (referent)	1.00
3–4	1.37 (0.66–2.84)
5	2.26 (1.04–4.90)

Abbreviations—Confidence Interval (CI)

^a^Odds ratios calculated from multiple logistic regression analysis and adjusted for all other variables listed in the table. These variables were independently associated with the career satisfaction in bivariate analyses and included here if the p-value from the bivariate analysis was p<0.25.

The multiple logistic regression model for perceived competitive salary contained five variables (Table 4). As with career satisfaction, perceived salary competitiveness was lower among those whose race/ethnicity was other than white, although this did not reach statistical significance. Persons in government or university/other research institutions also had lower perceived salary competitiveness (with a significant difference for government workers), as did length of time with current employer of 5–9 or ≥10 years. Having 3–4 leadership positions was associated with a greater likelihood of perceiving one’s salary as competitive. Fellowship cohort and sex were not associated with perceived salary competitiveness.

**Table 4:** Relative Odds of Selected Factors for Perceived Salary Competitiveness among Former Postdoctoral Fellows.

Factor	Odd Ratio and 95% CI^a^
Age	
<40 (referent)	1.00
40–49	0.58 (0.28–1.22)
≥50	0.75 (0.30–1.90)
Race/Ethnicity	
White, non-Hispanic	1.00
Other	0.51 (0.25–1.03)
Current Employer	
Other (referent)	1.00
Government	0.33 (0.11–0.98)
University/research institute	0.40 (0.15–1.08)
Years in Current Job	
<5 (referent)	1.00
5–9	0.31 (0.15–0.65)
≥10	0.37 (0.16–0.87)
No. of Leadership Roles	
0–2 (referent)	1.00
3–4	2.83 (1.25–6.42)
5	1.59 (0.72–3.51)

Abbreviations—Confidence Interval (CI)

^a^Odds ratios calculated from multiple logistic regression analysis and adjusted for all other variables listed in the table. These variables were independently associated with the perceived salary competitiveness in bivariate analyses and included here if the p-value from the bivariate analysis was p<0.25.

## Discussion

This study of predominantly Ph.D.-educated scientists in postdoctoral training positions in cancer prevention from 1987–2011 included individuals spanning a large range of employment history, from those nearing retirement to those just starting their careers. Overall, slightly more than 60% were very or extremely satisfied with their careers, with more than 70% perceiving their current salaries as competitive. There was some variation in career satisfaction, and perceived salary competitiveness, however, by selected demographic and other characteristics.

Career satisfaction was rated as “very” or “extremely” satisfied by 61% of this study population. Here career satisfaction was queried with regard to overall satisfaction with one’s career trajectory. Given the time and resources dedicated to postdoctoral training in biomedical research, it is reassuring that our study of career satisfaction, and other studies of current job satisfaction of predominantly PhD-trained populations from a wide range of scientific disciplines and across different employment sectors, report these individuals being generally highly satisfied.

While this estimate cannot be directly compared with findings from previous research that assessed satisfaction with one’s current job, it is similar in magnitude to prior studies of scientists. For example, in 2006, 64% of U.S. life scientists reported being extremely or very satisfied with their current job [32]. In the 2013 NSF SDR, 48% of US citizens who were scientists working in the United States reported being “very” satisfied (1 on a 4 point scale) with their current position [15]. A 2010 survey in the scientific journal *Nature* of scientists in different countries assessed overall current job satisfaction based on a composite measure of 8 factors, and used a 10-point decimal scale ranging from 0.0–1.0. They reported an overall satisfaction score for U.S. scientists of 0.628, or approximately 63% [33].

Given the absence of data on career satisfaction or perceived salary competitiveness in general, it is difficult to find comparisons with related fields or areas with long training periods. One such related area could be practitioners in preventive medicine disciplines, including public physicians or nurses. A 1997 study of U.S. preventive medicine physicians completing general preventive medicine residencies from 1979–1989 found that 88% reported high or very high overall levels of job satisfaction for their current position [34]. Current job satisfaction studies of public health nurses in three different states, based on scaled response categories, all reported an overall moderate level of job satisfaction [35–37]. Salive found a current salary satisfaction level for U.S. preventive medicine physicians of 60% [34]. In contrast, Juhl et al’s 1993 study of rural public health nurses in one Midwestern state found a current salary satisfaction level of 2.02 (on a 1–5 scale, with 5 being highest score)—this was the lowest score found across nine dimensions of job satisfaction for this population [35].

Even among this population of mostly satisfied individuals, there were factors inversely associated with career satisfaction or the perception of salary competitiveness. In univariate analysis, alumni of the CPFP program reported higher career satisfaction levels than NRSA/F32 awardees but these differences did not remain when career satisfaction was adjusted for other demographic and employment factors. Overall, the majority of respondents from each program reported high career satisfaction. In multivariate analysis, factors associated with career satisfaction or perception of salary competitiveness differed across the two outcome measures, but most were consistent with prior literature. Satisfaction with progression of one’s career to-date was inversely associated with age 50 years or more. Studies focusing on job satisfaction in general, not limited to the biomedical sciences or those with advanced degrees, have demonstrated a non-linear and perhaps U-shaped pattern of job satisfaction with the highest levels of satisfaction reported by those early in their careers or nearing retirement [38]. Among academic faculty, the most often studied career sector of biomedical scientists, it is also known that mid-career Associate Professors report the lowest levels of career satisfaction [10, 39]. Therefore, this finding of age differences, along with the finding that perception of salary competitiveness was lower among individuals who were employed in their current position 10 years or greater, may reflect the findings of these other studies and suggests that this career stage may be an area in which to target further career development opportunities.

In addition to age, identifying as a race or ethnicity other than White, non-Hispanic was associated with lower satisfaction with one’s career trajectory. While these findings are consistent with prior studies [7, 15, 40, 41], it is important to know when interpreting these data that race/ethnicity categories were collapsed in the analytic data set due to small cell sizes and concern about revealing personally identifying information. These cells cannot be disaggregated, but we do know individuals who self-identified as Asian comprise the largest part of the White, non-Hispanic population included here. All respondents were also U.S.citizens since this is an eligibility requirement for both the CPFP and NRSA/F32 programs.

Although there have been many studies of scientists’ actual or reported salaries, to our knowledge there are few studies of salary satisfaction. A 2006 survey of U.S. life scientists who were members of the American Association for the Advancement of Science (AAAS) reported higher career satisfaction among those who rated their salary as excellent [32]. Respondents to a 2010 international survey of scientists about their current jobs reported a salary satisfaction score of 0.511 (~51%) [33], which was much lower than we found for perceived salary competitiveness. Our finding of a lower perception of salary competitiveness among those employed in government settings stands out as in contrast to data reported from other studies, which indicate comparable or higher levels of salary satisfaction among government employees compared to those employed in other sectors [11, 15].

Positive factors associated with both career satisfaction and perception of salary competitiveness included having more leadership roles and, for career satisfaction alone, higher salary. Studies of career satisfaction in general have shown salary and career satisfaction are tightly linked [6, 15, 32, 33, 40], as are career satisfaction and opportunities for career growth [15, 40]. The data presented here repeat these same patterns with both salary and leadership opportunities, as a potential marker of opportunities for career growth. Future studies with this population will examine the associations of career satisfaction and salary competitiveness with leadership roles in more depth.

The strengths of this survey and study design include the ability to query individuals across multiple cohorts, employment sectors, and disciplines to gain an overall picture of career satisfaction post-training for those who completed postdoctoral fellowships in the biomedical sciences, and who had a particular interest in cancer prevention-related topics during their postdoctoral training. Although there were differences by postdoctoral training program in the univariate analysis, these differences did not remain in the multivariate analysis. Females were over 63% of the respondents, yet contrary to other studies of career satisfaction particularly in academic careers [6, 13, 19, 20, 42], we found no difference in satisfaction or perception of salary competitiveness by gender. There were also no differences in career satisfaction or perception of salary competitiveness by biomedical scientific discipline.

There were some notable study limitations. Although response rates were relatively high for an internet-administered survey [43, 44], respondents may have differed from nonrespondents, and we had limited demographic data in which to evaluate this. It is also possible respondents differed from nonrespondents with regard to career satisfaction or salary perceptions. We also saw response rates vary by program. This may be due in part to the purpose of the survey being to understand outcomes of the CPFP. The response rates may reflect the CPFP alumni having greater interest in providing feedback to the program where NRSA/F32 awardees would not have the same motivation. In addition, a single survey item each was used to measure career satisfaction and perceived salary competitiveness, which may have resulted in higher or lower estimates than would have been obtained using multiple survey items [45–47]. Data were also obtained from self-reports, thus the validity of responses about current employment (e.g., salary, length of time in current position) and other factors could not be assessed.

In total, these data provide important insights into the post-training employment of predominantly PhD-trained scientists who conducted postdoctoral research in cancer prevention-related areas, and these methods could be used to explore career outcomes relative to postdoctoral research participation in other scientific fields. These data also provide reasons for optimism by demonstrating most individuals in this study are satisfied with their career trajectory to-date and perceive that they are being fairly compensated. Further, given recent studies reporting both the varied career interests of biomedical scientists following postdoctoral training and available opportunities [48–53], it is reassuring that the levels of high career satisfaction were consistent across employment sector explored here. There are also ongoing efforts to collect and aggregate more career outcome data at a national level to understand how PhD and postdoctoral-training influences individual earnings and the collective biomedical research economy [26]. All of these efforts at both a national and local level will enable graduate students and postdoctoral fellows to make better informed decisions about their future career paths and for contemporary trends in employment to be assessed at a finer level.
